# Hexagonal germanium formation at room temperature using controlled penetration depth nano-indentation

**DOI:** 10.1038/s41598-018-38440-3

**Published:** 2019-02-07

**Authors:** Ghada Dushaq, Ammar Nayfeh, Mahmoud Rasras

**Affiliations:** 1 Electrical and Computer Engineering, New York University, Abu Dhabi, UAE; 2 Electrical Engineering and Computer Science, Khalifa University, Abu Dhabi, UAE

## Abstract

Thin Ge films directly grown on Si substrate using two-step low temperature growth technique are subjected to low load nano-indentation at room temperature. The nano-indentation is carried out using a Berkovich diamond tip (R ~ 20 nm). The residual impressions are studied using *ex-situ* Raman Micro-Spectroscopy, Atomic Force Microscopy combined system, and Transmission Electron Microscopy. The analysis of residual indentation impressions and displacement-load curves show evidence of deformation by phase transformation at room temperature under a critical pressure ranging from 4.9GPa–8.1GPa. Furthermore, the formation of additional Ge phases such as r8-Ge, hd-Ge, and amorphous Ge as a function of indentation depth have been realized. The inelastic deformation mechanism is found to depend critically on the indentation penetration depth. The non-uniform spatial distribution of the shear stress depends on the indentation depth and plays a crucial role in determining which phase is formed. Similarly, nano-indentation fracture response depends on indentation penetration depth. This opens the potential of tuning the contact response of Ge and other semiconductors thin films by varying indentation depth and indenter geometry. Furthermore, this observed effect can be reliably used to induce phase transformation in Ge-on-Si with technological interest as a narrow band gap material for mid-wavelength infrared detection.

## Introduction

Epitaxial Growth of Ge-on Si platform has found a tremendous research interest in novel high-speed electronic and photonic devices^1–4^. It is very critical for nanoscale devices such as Fin-Shaped Field Effect Transistor (FinFETs) and Nanowire Field Effect Transistor (NW-FETs) to employ channel materials such as Ge with high electron and hole mobility^5,6^. Furthermore, Ge narrow band-gap allows for efficient infra-red detection in opto-electronic devices^7,8^. However, bulk Ge is mechanically softer than Si, and Ge/Si system can have residual strain introduced by the integration process. Residual strain in Ge thin films on Si consists of three components: (i) the 4.2% lattice mismatch strain (ii) thermal misfit strain (coefficient of thermal expansion CTE), and (iii) threading dislocation or void creation as a form of strain relief. This strain could raise concerns over the mechanical stability of Ge-on-Si layer. However, under appropriate internal (or residual) stresses, the mobility of charge carriers and thus the speed of the device can be significantly increased^9,10^. Furthermore, the presence of residual in-plane tensile strain leads to a decrease in the direct and indirect Ge band gap^11,12^. Therefore, understanding the mechanical properties of Ge/Si system is crucial and of significant interest in modern technology.

Instrumented nanoindentation is a widely utilized technique and a well-controlled method for measuring the mechanical properties of bulk materials and thin films^13–15^. This technique allows for understanding the mechanical response of materials by applying a highly localized mechanical deformation. Moreover, controlled growth of self-assembled nanostructures can be performed in a complete physical method^16–18^. Indentation with conical or spherical diamond tips has been carried out on bulk Si and Ge to study their deformation mechanisms. In silicon it was found that indentation-induced phase transformation is the dominant deformation mechanism^19,20^. However, substantial differences in the deformation mechanisms in Ge have been observed. Two different deformation processes have been found to occur at room temperature. First, deformation controlled by the formation and propagation of defects such as slip and twinning^21,22^. This deformation is observed when indenting with both spherical and Berkovich tips. Second, deformation via phase transformation, in which a variety of phases have been reported on unloading such as simple tetragonal structure (st12-Ge), traces of rhombohedral structure (r8-Ge), and body centered cube (bc8-Ge), as well as amorphous (a-Ge)^23^. The phase transformation in Ge appears to only occur during extreme loading conditions including fast loading rates, high loads, and very sharp tips^24–29^. Furthermore, at lower temperature Ge shows similar behavior to room temperature Si, where a phase transformation below 0 °C has been reported^30^. It is clear that phase transformations in Ge at room temperature even under such extreme conditions is random and does not have a 100% probability of occurring compared to Si, which has been extensively studied to undergo a pressure-induced phase transformation at room temperature^31,32^.

There have been only few previous studies on the transformation paths of Ge-on-Si system^33,34^. This includes the induced structural changes in the films with varying indentation process conditions. In fact, the Ge-films has higher complexity compared to bulk Ge. In this case the film thickness and the substrate effect should be taken into consideration^34,35^. Thus, the indentation depth should not involve more than 10–25% of the film thickness^36–38^. Furthermore, the preferred deformation mechanism is highly affected by the preparation method. These include the morphological, film constituents and possible impurity^21,34^. Therefore it is critical to understand the induced structural changes of Ge-on-Si under a point indenter.

In this work we report nanoindentation of Radio-Frequency Plasma Enhanced Chemical Vapor Deposition (RF-PECVD) Ge films directly grown on Si substrate. The growth is based on low temperature two-step technique which is described in our previous work^39^. In the present work we focus on Ge on Si films which have been grown using RF-PECVD at temperature <500 °C. This system has an advantage of depositing Ge at low temperature of 400 °C. Furthermore, the RF-PECVD reactor is capable of promoting the nanocrystal growth at low temperatures via plasma contribution. Results show that the measured threading dislocation density (TDD) of 700 nm thick Ge films grown at 500 °C is ~1 × 10^6^ cm^−2^, which paves the way to achieve high quality monolithic integration for the next generation of electronic and photonic devices. Additionally, Electron Dispersive Spectroscopy (EDS) analysis carried out on the film does not show impurities like (H, O, and C) in the grown layer, although the system is not an ultra-high vacuum system (UHV). The film shows transport proprieties dominated by holes with carrier density of ~5 × 10^17^ cm^−3^ and hole mobility <75 cm^2^·(V·s)^−1^ as a function of magnetic field. To explore more potential application, the films are subjected to depth controlled nanoindentation, where a penetration depth of ~150 nm, 210 nm, 290 nm, and 350 nm have been performed at room temperature. The phase transformation in Ge films at low load is confirmed by analyzing the load-displacement curves and the characterization of the indents using Raman micro spectroscopy with Atomic Force Microscopy (AFM) cantilever and cross-sectional transmission electron microscopy (XTEM). The Results are explained in terms of the spatial distribution of the incused pressure in Berkovich tip, threading dislocation density variation with film thickness, stress-strain in the film, films preparation method and impurity level and the effect of Si substrate. The observed transformation of Ge to hexagonal (hd-Ge) phase beneath the indents is of technological interest due to its predicted narrow direct band gap <0.55 eV, which makes it ideal for infrared detection. Furthermore, tailoring the film response as a function of indentation penetration depth can offer a novel means of inducing phase transformation at room temperature for a wide range of semiconductor films.

## Results

**Figure 1:**
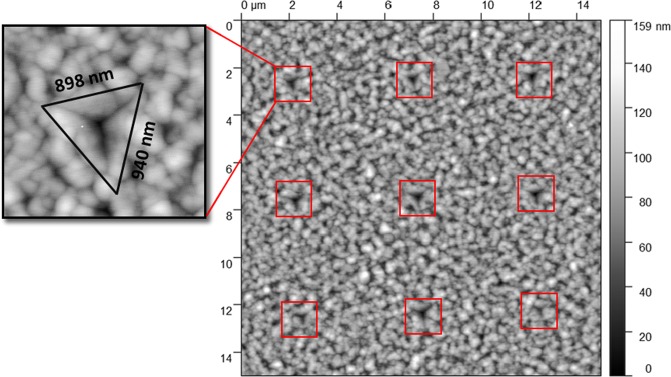
15 × 15 µm^2^ AFM micrograph of indentation array performed using Berkovich indenter at room temperature, and zoom in image of indent geometry showing *x*, y extrusion attributed to 150 nm indenter depth.

**Figure 2:**
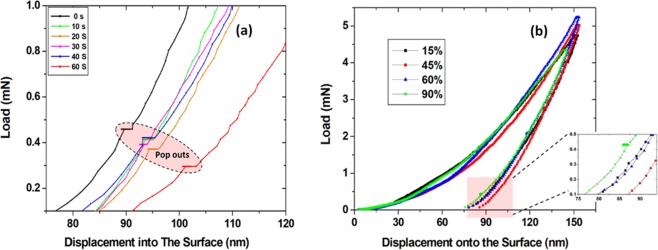
Load-displacement (*P*-*h*) curves in depth control mode of 150 nm. (**a**) Typical unloading curves observed when holding time between loading and unloading is varied between 0s–60s at fixed unloading percentage of 90%. (**b**) The effect of changing unloading percentage on the existence of pop out event in unloading curves, where no pop out is observed for unloading percentage less than 75%.

Figure 1 shows AFM scan image of the residual impression of the indents array performed at 150 nm indentation depth. Using simple calculation, the projection area can be calculated based on the indents shape and the Berkovich indenter geometry^13,40^. The 150 nm is the maximum tip penetration depth defined by the user (we created indentation with different maximum penetration depth of 210 nm, 290 nm, and 350 nm in a depth control mode of the Agilent Technologies Nano Indenter G200). The area of contact at full load is determined from the known angle or radius of the indenter. The projected area function A (h_c_) for the perfect Berkovich indenter is given by A_c_ = 24.5 h_c_^2^, h_c_ is the contact depth which is not the user defined of maximum penetration depth due to the elastic recovery of the film^13,40^. To find the projection area we extracted the contact stiffness *S* (slope of load-displacement (*P*-*h*) curves, shown in Fig. 2), final depth h_f_, maximum load (P_max_) and the maximum depth h_max_ (150 nm) from the *P*-*h* curves and inserted them in this equation (h_c_ = h_max_ − 0.75x(P_max_/S)).

The approximate value of the calculated projection indents area with 150 nm µm maximum penetration depth is 0.55 µm^2^. This value is close to our measured area of the residual imprints.

The unloading part of the load-displacement (*P*-*h*) curves at an indentation depth of 150 nm for different holding times and unloading percentile are shown in Fig. 2a,b. The holding time between loading and unloading is from 0 to 60 s with a corresponding maximum load of 4.5 mN and 2.75 mN, respectively. The average contact pressure values at the early stages of indentation are high and then rapidly decreases during the loading with the penetration displacement. After that, it quickly reaches a more or less constant value. Using the calculated area value, this means that the induced contact pressure of the tip is varied between 4.9 GPa to 8.1 GPa. It is important to point here that the pressure distribution is not uniform since the contact effective shape changes at different points inside the indents since the tip is of pyramid-shaped (area change), more details will be found in the discussion section. The loading curves of 49 indents are analyzed, some of them are featureless where no pop-in events have been observed, however others exhibit pop ins in the loading part. On the other hand, pop-out events were clearly apparent in all the unloading curves. These events exhibit almost the same size with slight variation in position, where for 0 s and 60 s holding times, the pop-outs were observed at ~0.5 mN and ~0.3 mN loads, respectively. Figure 2b shows the loading and unloading curves of indentation behavior while changing the percentage of unloading (partially released after loading) in the depth control mode of 150 nm. Similarly, some of the loading curves were featureless, however the unloading percentage affects the appearance of pop out event in unloading curves. As can be seen clearly from Fig. 2b, for unloading percentage less than 75% no pop out event was observed.

The pop-in and pop-out events in *P*-*h* curves are usually indicative of the type of mechanical deformation process in the Ge film. A pop-in event (partially observed in our *P*-*h* curves) occurs as an indication of slip, twinning and shear activity^21,30^. However, a pop-out event is linked to the formation and nucleation of other Ge crystalline phases such as st12-Ge, r8-Ge and hd-Ge^24–27^. The primary reason of those pop-out events is the sudden change of a discrete volume of material from high density phase to low density phase. For instance, bulk Ge is known to phase transform to the metallic beta-tin structure (b-Sn-Ge) at 10 GPa^41,42^.

**Figure 3:**
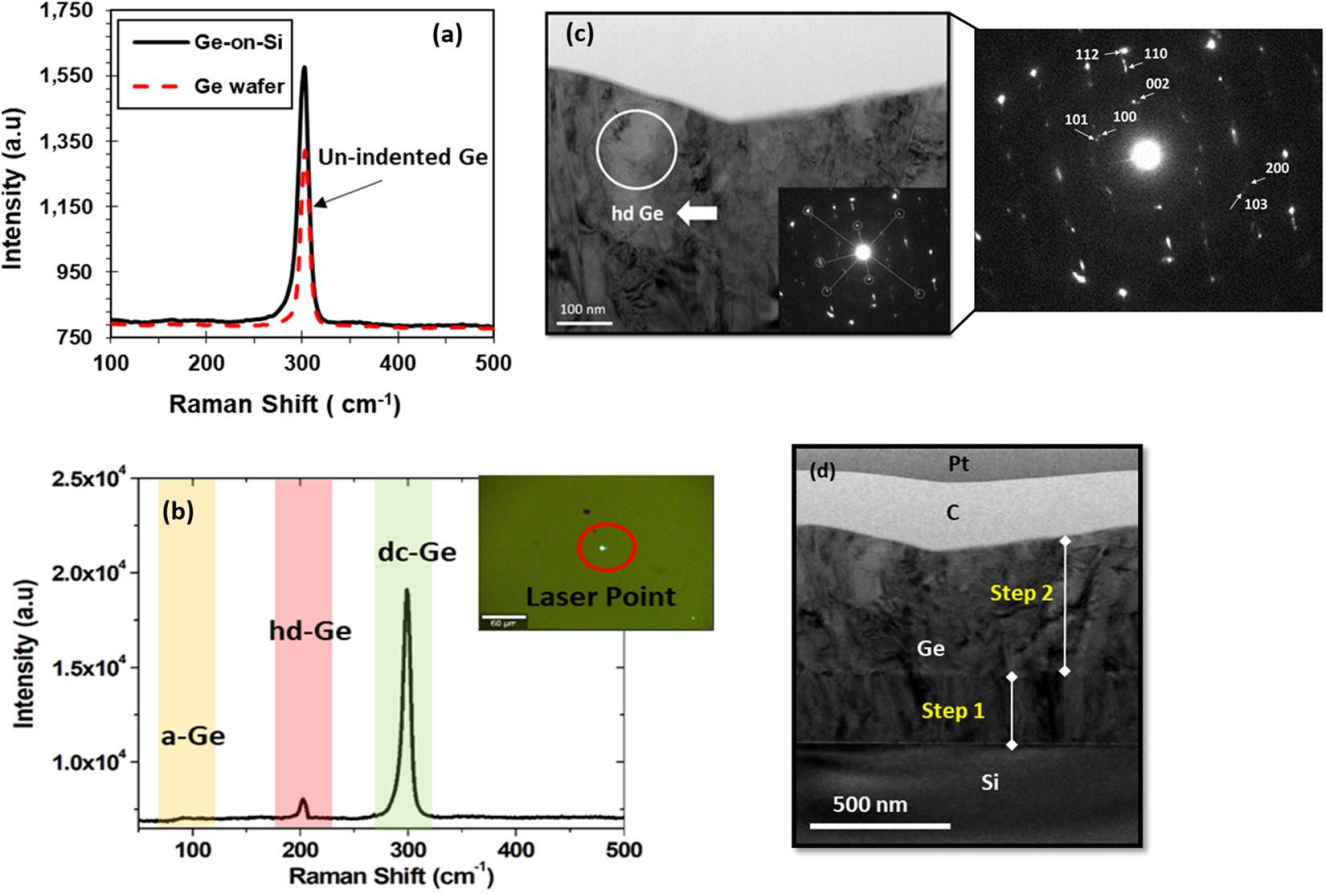
Micro-Raman spectra and bright field XTEM micrographs. (**a**) Raman spectrum of un-indented film and Ge wafer. (**b**) Raman spectrum of indented Ge film. (**c**) XTEM image of indentation impression carried out in depth control mode for indentation depth of 150 nm at room temperature. The selected area diffraction pattern (SADP) are collected in three region (right-center-left) beneath the indent, the SADP shown represents the region in the circle and its indexing (**d**) low magnification TEM image showing the entire indent region.

In order to further study the microstructure changes in the vicinity of indent both confocal Raman spectroscopy and cross section TEM characterization are carried out. Figure 3b shows the Raman spectrum of the indentation impression, where the Raman laser spot is aligned with indents location using *in-situ* AFM scan. Three Raman spectra are displayed, two for un-indented bulk Ge and Ge-on-Si films (Fig. 3a) and the other is taken at the indent position (Fig. 3b). The spectra from un-indented regions of the films feature a single peak at 301 cm^−1^, a characteristic of diamond cubic (dc-Ge). However, the Raman spectrum of indented Ge exhibits both a (dc-Ge) peak centered at ~301 cm^−1^ and an additional peak at ~202 cm^−1^. This extra peak with other peaks position at 86 cm^−1^, 94 cm^−1^, 202 cm^−1^, 225 cm^−1^, and 249 cm^−1^ are identified to be linked to the presence of the r8 phase of Ge^24–27,43,44^. It was reported that unlike Si BC8 and R8 phases which coexist stably under ambient conditions, germanium phases R8 and BC8 are observed to transform to a hexagonal diamond phase under ambient pressure and temperature^23,43^. Therefore, the r8-Ge phase is not stable and was observed to transform to hd-Ge at room temperature. The Raman spectrum of indents is obtained after several days of indentation that led to the observation of hd-Ge rather than r8-Ge. Additionally, by mapping the indentation area using 532 nm laser light and a gratings of 1800 gr/mm we observed that the peak intensity at 202 cm^−1^ varies between one indent to another, this might be attributed to the variation in the fraction of the transformed phase.

To further clarify the end phases present in the indented zone, bright-field TEM micrograph was carried out, data is shown in Fig. 3c,d. No cracks, twinning or crystal defects are observed in the underlying region of the indentation. The selected area diffraction patterns (SADP) are taken from the center and the edges of the indented zone. The measured d-spacings of the region in white circle of Fig. 3c are described in Table 1, and the indexing are performed in Fig. 3c. The values are compared with the d-spacings of hd-Ge calculated after the unit cell given by Brazhkin *et al*.^45^ of a = 3.94 Å, c = 6.55 Å. There is an excellent agreement between the measured and calculated d-spacings which confirms that the dominant end phase within the transformed zone is hd-Ge. However, the pristine Ge SADP d-spacings (not shown) match with DC-Ge diffraction spots using a unit cell with a = 5.64613 Å. The d-spacing of DC-Ge and hd-Ge are very close and it is hard to distinguish them. However, the presence of all other hd-Ge spots that do not overlap with DC-Ge diffraction spots indicates that our end phase is hd-Ge. Additionally, diffused cycles are observed in the SADP from the edges and center of the indents which can be due to the amorphous phase in the zone. We believe that in the Raman spectrum the laser beam does not penetrate below the indent (penetration depth <30 nm) due to the large optical absorption coefficient of Ge at the excitation wavelength of 532 nm, thus the amorphous phase contributes slightly to the measured Raman spectrum (broad peak around 100 cm^−1^)^30,43,46^. Based on this we suggest that the fraction percentage of a crystalline hd-Ge end phase might be enhanced at higher temperature. Therefore, further study at higher temperature is needed.

**Table 1:** The measured d-spacings for hd-Ge from SADP diffraction spots shown in Fig. 3c.

*h k l*	Measured *d* (A)
100	3.38
002	3.26
110	1.97
200	1.73
112	1.67
103	1.80

The values are compared with the d-spacings of hd-Ge calculated after the unit cell given by Brazhkin *et al*.^45^ of a = 3.94 Å, c = 6.55 Å. The values are observed at right-center-left of the indent at 150 nm indentation depth.

**Figure 4:**
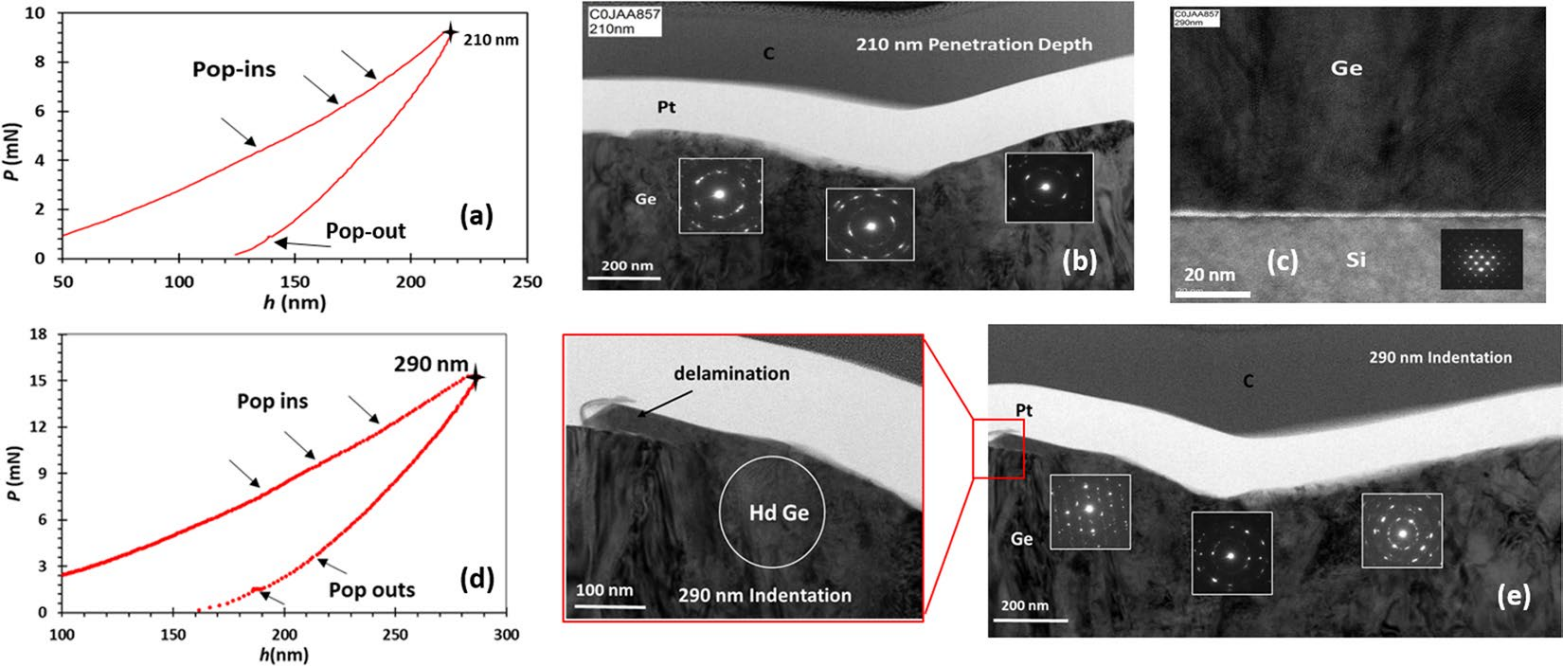
*P*-*h* curves and bright field XTEM micrographs of indents at different penetration depth. (**a**) *P*-*h* curves of indents at 210 nm depth depict the pop-in events in the loading part. (**b**) XTEM micrograph of indent at 210 nm depth and SADP at the left-right edges and the center of the region beneath the indent. (**c**) TEM cross section of Si/Ge interface and SADP of Si in a direct line with indentation center. (**d**) *P*-*h* curves of indents at 290 nm depth depict the pop-in and the extra pop out events in the loading and unloading curves. (**e**) XTEM micrographs of indent at 290 nm depth and SADP at the left-right edges and the center of the region beneath the indent, the zoom in image focuses on the delamination happened at this depth.

To further understand the dependence of the preferred deformation mechanism on the tip penetration depth, its contact configuration and the stress distribution, a different indentation penetration depths of 210 nm (30% of film thickness), 290 nm (41% of film thickness) and 350 nm (50% of film thickness) have also been carried out. The *P*-*h* results for all Ge-on-Si samples with different penetration depths showed similar behavior and elastic recovery, but differed significantly in terms of qualitative features of the curves. Most notably, the tendency for pop-in events occur on the loading curves which are related to shear activity. Additionally, extra pop outs in the un-loading curves were apparent in the 290 nm sample. Figure 4(a,d) show the *P*-*h* curves from indents at penetration depth of 210 nm and 290 nm, respectively. For 210 nm depth, with maximum load of ~9 mN, a pop in events were observed in all loading curves. Likewise, loading curve of 290 nm penetration depth tends to feature pop-in events at a maximum load of ~14 mN, as well as some extra pop-outs in the un-loading curve compared to 150 nm and 210 nm samples. The pop-in events are an indicator of the occurrence of plastic deformation that might lead to phase transformation in the severely compressed region^47,48^.

XTEM micrographs show the extent of structural changes and phase transformation in more detail. Figure 4(b,e) shows micrographs of indents at 210 nm and 290 nm depth, respectively. No clear cracks, twinning or crystal defects are observed in the underlying region of the indentation. The SADP from the left-right edges and the central region of the indented zone show different transformation behavior. The transformed regions of the Ge are distinguishable by the darker contrast in the XTEM micrographs. The SADP at both penetration depths contain some diffused cycles which is linked to a-Ge phase with small grains of polycrystalline Ge. Additionally, the measured d-spacings of the region in the left-right and center of 210 nm and 290 nm samples are compared with the values in Table 1 for hd-Ge, some d values are in good agreement with hd Ge d spacing, however not all d values are observed in their diffraction pattern as seen in the 150 nm case.

**Figure 5:**
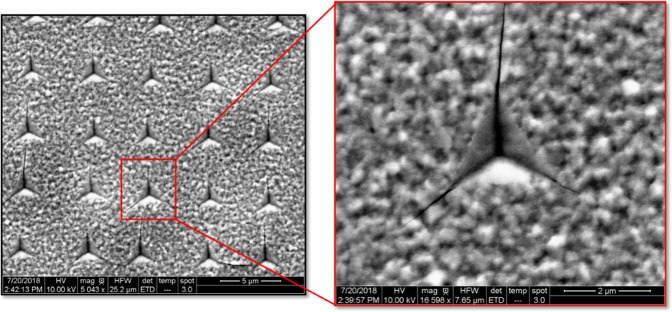
SEM micrographs of indents performed on Ge-on-Si films with tip penetration depth of 350 nm.

Figure 4(e) depicts a delamination which has been occurred at penetration depth of 290 nm. The Ge has the appearance of having flowed to the surface. Thus, indentation with deeper penetration depths were performed to understand the trend of the film fracture. Figure 5 shows SEM micrographs of indents with tip penetration depth of 350 nm (50% of the film thickness) and maximum applied load of ~20 mN. There is visible micro cracks around the corners of the indents and uplift of Ge material in other locations of the sample (not shown). Therefore an applied load above 20 mN results in a film fracture and damage. For this reason we limited the study on the deformation mechanism as a function of tip penetration depths to loads less than 20 mN.

## Discussion

**Figure 6:**
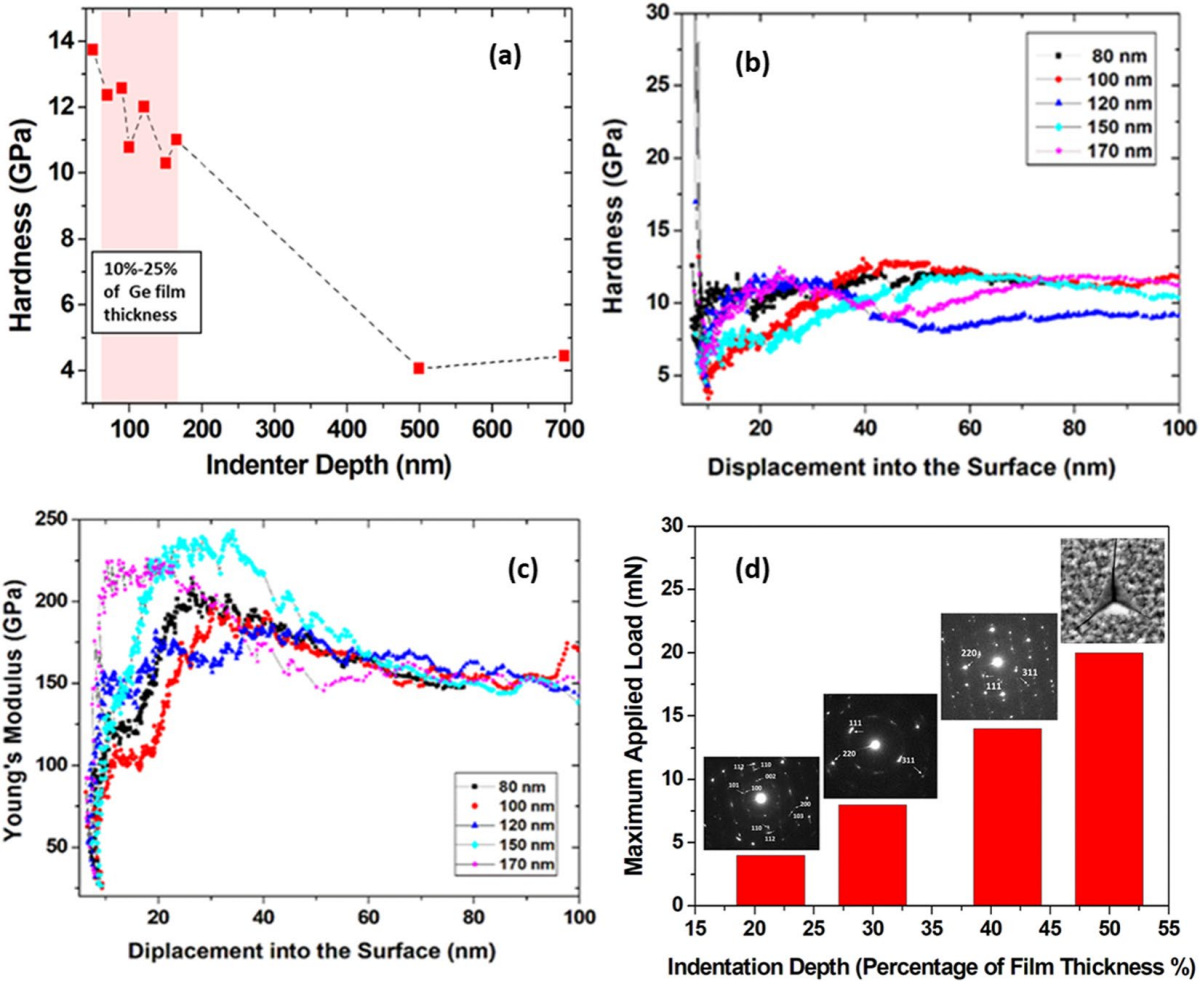
Hardness and maximum applied load values as extracted from *P*-*h* curves (**a**) hardness as a function of tip penetration depth (point analysis) (**b**) Ge film hardness values as obtained from continuous stiffness method (CSM) at different depth of 80 nm, 100 nm, 120 nm, 150 nm, 170 nm (**c**) Young’s modulus of Ge films as obtained from continuous stiffness method (CSM) at different depth of 80 nm, 100 nm, 120 nm, 150 nm, 170 nm (**d**) pathways of phase transformation in Ge-on-Si films as a function of tip penetration depth.

In this section, we discuss the underlying mechanism of the phase transformation in the Ge films. It is known that bulk DC-Ge needs ~10 GPa to phase transform to the metallic beta-tin structure (β-Sn-Ge)^41,42^. Also, it is difficult to change its phase under indentation loading and deformation by slip where twinning is more favored^29,30^. Furthermore, the magnitude of the pressure required to induce transformation in Si and Ge and their hardness is similar. However, from our data, Ge-on-Si system favors phase transformation at room temperature and under low indentation load with a maximum pressure values between 5GPa–8GPa. This result suggests that residual stresses in the Ge film due to the lattice mismatch and differences in thermal expansion during cooling in the deposition process play an important role in the observed phase transformation. The XRD data and Raman spectra performed previously on pristine Ge-on-Si film show that films are in tensile configuration with respect to the Si substrate^39^. Based on this observation, we expect films softer than bulk Ge due to the tensile strain. Therefore, it is worth examining the mechanical properties of the films such as the hardness compared to bulk Ge. Figure 6(a) shows the hardness values obtained from films loaded at different penetration depths. The measured values are based on the measurements of the contact stiffness vs. depth of the indentation. It employs a calibrated indenter tip area function and a prior knowledge of the elastic properties of the film and the substrate following. Therefore, the basic definition of hardness as the load divided by the projected contact area is used in Fig. 6a^13,40^. It is clear from the figure that the films are softer than our measured hardness value for bulk Ge under Berkovich indenter tip (12.9 ± 0.6) GPa. In this case the hardness values are considered when the indentation depth is limited to a small fraction of the film thickness (typically 10–25%)

In practice, we believe that effects associated with both the surface roughness and the elastic modulus mismatch between the Ge film and the Si substrate limit the applicability of the Oliver and Pharr method. For instance, our Ge/Si system represents a soft films on hard substrates, thus we expect a film material piles up against the sides of the indenter during indentation, causing a larger contact area than is predicted by the calibrated A_c_(h_c_). Thus, the Oliver and Pharr approach overestimates the hardness when pile-up occurs.

Therefore, there is a need for another technique to report the hardness values of our Ge films over a range of indentation depths. Additional indentation tests were performed using continuous stiffness measurements (CSM). In this method the contact force and penetration are measured continuously as a hard indenter is pressed in to contact with and then withdrawn from the Ge film^49^. Therefore, the mechanical properties of the Ge film is measured as a continuous function of distance into the surface (depth profile of properties). This is expected to give a more robust and accurate hardness values. Figure 6b,c show the hardness and the Young’s elastic modulus of 700 nm Ge-on-Si using CSM method at different depths of 80 nm, 100 nm, 120 nm, 150 nm, 170 nm.

As can be seen in Fig. 6b,c the hardness results are generally not as constant as the modulus results. Additionally, the measurements are stable till 100 nm indentation depth after that it shows instability where values can’t be reported. Results of hardness values have been extracted from the stable part in the curve of CSM tests. Still, hardness values less than that of bulk Ge are observed.

This is consistent with the tensile configuration observed in the film. Another way to view the observed softening effect is to observe misfits and threading dislocations which can significantly reduce the strength of the material. Previously, it was shown that the hardness is proportional to the density of atomic bond strength in covalent semiconductors^50,51^. Thus, the hardness value is lowered in the presence of misfit dislocations and crystal defects. Moreover, threading dislocations and voids in a structure could significantly affect the deformation behavior. Our previous TEM studies of Ge/Si interface shows that the first 50 nm in the growth indicates a significant mismatch where the crystal defects extending from the interface toward the surface (high TDD close to Ge/Si interface and less toward the surface). This explains the changes in hardness values with penetration depth. Furthermore, the observed a-Ge phase in some regions of 210 nm and 290 nm samples might trigger a crystal collapse due to the interaction of the indenter with pre-existing defect, this has been seen previously^25^. Similarly, this can be correlated to the *P*-*h* curves as the following: when the indenter tip is in contact with the pre-existing threading dislocations, it generates a sudden increase in plasticity which can be observed by the elbow (change in slope) and pop-ins, pop outs in the *P*-*h* curves. Additionally, we believe that microstructure of the films, its impurity level, the preparation method and the deposition condition (pressure, temperature, Rf power, gas precursor) are all affecting film hardness^21,34^. Thus, further examination of Ge films with different hydrogen content would be of interest to observe its effect on the phase transformation path ways under Nano-indentation depth sensing method.

**Figure 7:**
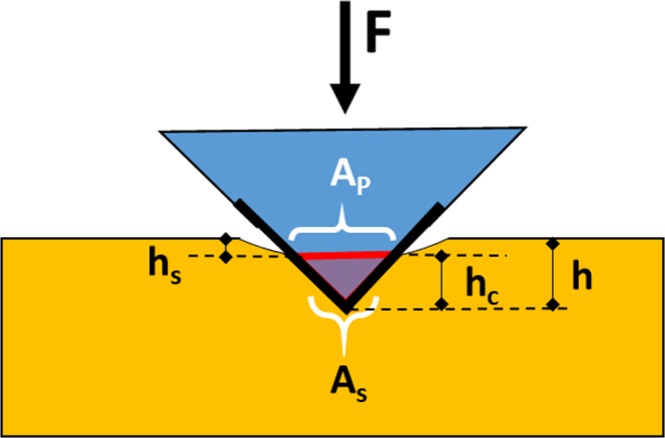
Schematic of Berkovich indenter showing the indentation projection and surface areas and depth parameters.

To further elucidate on the underlying mechanism of the phase transformation as a function of tip penetration depth, a closer look at the effective tip shape is needed. The mean contact pressure is usually determined from the measured contact depth of penetration, *h*_*c*_ (see Fig. 7), such that the projected area of the contact and surface area for a Berkovich tip with face angle of 65.3° are given by: *A*_*proj*_ = 24.49 *h*_*c*_^2^*, A*_*surf*_ = 26.98 *h*_*c*_^2^, respectively^40^. Based on this, the contact pressure is defined as *P*/24.49 *h*_*c*_^2^ where *P* is the applied load. Previous studies based on theoretical and molecular dynamics simulation for Berkovich tips showed that the pressure is constant along the contact area and drops rapidly at the boundaries. However, the pressure distribution under the contact effective shape reveals a completely different contact pressure map^52–55^. The non-uniform distribution of the pressure (high pressure toward the boundaries of the contact area) can be understood from the tip effective shape.

In our experimental results the variation in the indenter depth modifies the interaction volume beneath the intended zone which results in different absolute maximum and minimum values of shear and hydrostatic pressure. We can conclude that the variation of the pressure spatial distribution beneath the tip can contribute to the observed phase transformation modes in 150 nm, 210 nm and 290 nm samples as depicted in Fig. 6d. In the context of these findings hd-Ge phase is energetically favorable at low penetration depth since it is highly visible in 150 nm sample (21% of the film thickness). This means that the percentage of indentation from the overall film thickness is significantly affecting the pressure-induced phases observed in the film, particularly in lattice mismatched systems. Furthermore, we suspect that in our film, the indentation load applied to the film may have been partly absorbed by Si substrate and distributed over larger area. Therefore, the local stresses concentration beneath the tip is insufficient for slip and twin bands to occur. However, no phase transformation or shear plasticity are observed in the Si substrate as shown in Fig. 4c.

In the context of these findings, we can infer that thin films with harder substrate can hold phase transformation under moderate load. Further examination can be performed for Ge films grown on Ge substrate and softer substrate material like GaAs. Additionally, Ge-on-Si system resembles the behavior of room temperature Si, where deformation is dominated by phase transformation. Thus, we assume that unloading rate can affect the end phase of Ge-on-Si layers. Engineering the crystal phase with tip depth and geometry may offer a novel means of inducing phase transformation at room temperature in a wide range of semiconductor films.

In summary, the deformation mechanism and the transformation pathways in Ge-on-Si films subjected to nano-indentation at room temperature are investigated using a combination of micro-Raman, AFM and XTEM techniques. The Ge films are grown directly on Si substrate using two-step low temperature growth technique by RF-PECVD. Results show that Ge films exhibited pressure induced phase transformation deformation mechanism. This is evident by the formation of additional Ge phases such as r8-Ge, hd-Ge, and amorphous Ge as a function of indentation depth. A hd-Ge phase is observed in all performed indents with indentation penetration depth of 150 nm. Furthermore, the correlation between the inelastic deformation mechanism and the indentation penetration depth has been realized. It was seen that the non-uniform spatial distribution of the shear stress due to the pyramid-shaped tip plays a significant role in determining the end phase of Ge^52–55^. Finally, the observed phase transformation to hd-Ge (direct band gap <0.55 eV) and the possibility to tune the crystal phase via modifying indentation condition opens a new opportunity for on-chip laser on silicon and infrared detectors.

## Methods

### Sample preparation

700 nm Ge films have been grown on (001) Si substrate using low temperature two-step approach by RF-PECVD. In step one (low temperature (LT), high rate (HR)) the deposition was performed at 350 °C with 3 sccm flow of GeH_4_. This step acts as a seed film for the second growth. After this, step two (high temperature (HT), low rate (LR)) of the Ge deposition starts immediately without removing the wafer from the system. The Ge growth carried out at 500 °C with 1 sccm of GeH_4_. With these two steps one cycle of Ge growth is done. More detailed growth parameters and films characterization can be found in our earlier publication^39^. Before the indentation process, samples are cleaned using acetone, isopropanol and DI water to achieve high-quality Ge surface which is free from contamination and clear to start the nano-indentation.

### Material characterization

#### Indentation

The nano-mechnical response of the Ge films, in particular the displacement-load curves are probed using Agilent Technologies Nano Indenter G200 using diamond pyramid-shaped Berkovich-type indenter tip. The indenter tip has a nominal radius of about <20 nm with the pyramidal faces forming an angle of 65.3 with the vertical axis, this has been measured using AFM and HRTEM. The analysis of thin film mechanical properties is significantly affected by the tip rounding, and indenter geometries, since the known geometry of the indenter then allows the size of the area of contact to be determined. Since we are targeting films in nano meter scale grown on substrate it is very critical to choose very tiny radius of the indenter where the large tip radius has an effect on the Nano-mechanical response of the film at small loads. This was possible in Berkovich tips since the pyramid are more easily constructed to meet at a single point, rather than the inevitable line that occurs in the four-sided Vickers pyramid. The face angle of the Berkovich indenter normally used for nanoindentation testing is 65.27°, which gives the same projected area-to-depth ratio as the Vickers indenter. The tip radius for a typical new Berkovich indenter is on the order of 20–100 nm. This usually increases to about 200 nm with use.

**Figure 8:**
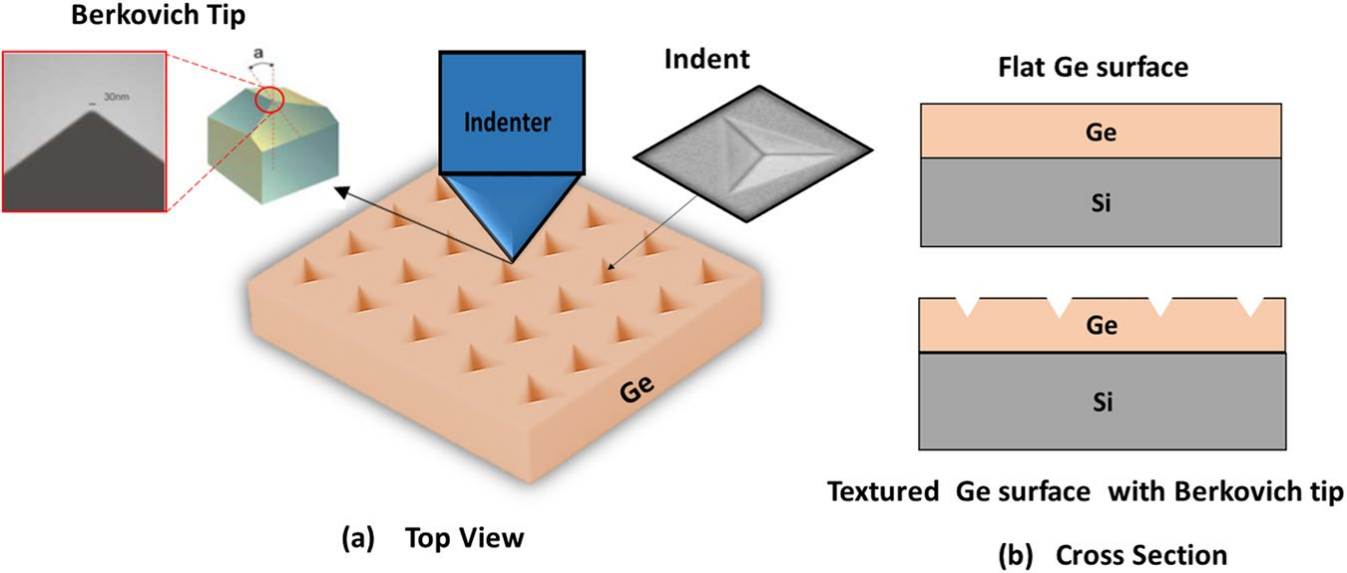
Indentation process (**a**) top view of Ge patterned surface with an SEM images of Berkovich tip and indents shape (**b**) side view of textured Ge surface.

An array of 49 (7X7) indents was performed in a single cycle automated indentation process with surface approach of 1 µm and fixed tip velocity of 10 nm/s. To avoid any substrate contribution, the indentation depth should be limited to 10–25% of the Ge film thickness. A maximum indentation depth of 150 nm was used to form inverted pyramidal impressions with lateral size of ~900 nm at the ambient temperature of 27 °C. Figure 8 shows a schematic of Nano indentation process on Ge-on-Si films using Berkovich diamond tip. Indentation with penetration depth of 210 nm, 290 nm and 350 nm have also been carried out, however we first focused on 150 nm depth (10–25% of the Ge film thickness) then we discussed other indentation depths. A loading and unloading rates of 0.27–0.57 mN.s^−1^ with holding time varying between 0–60 s have been used during the indentation process. Additionally, the tests were performed in which the load was partially released after loading, thus, the unloading percentage was modified between 15–90% of the maximum load.

#### Micro-Raman & AFM

The indentation impression was examined using Raman micro-spectrometry in addition to the AFM system.

The scanned images of indents using AFM are taken several days after indentation where no pre-surface cleaning (no HF removing GeO_x_) was performed prior to imaging. The air exposure creates surface oxidation due to the high growth rate of GeO_x_ (>0.2 nm/min)^56^. We avoid HF cleaning after indentation where it can etch Ge a little bit and affect the indent shape. Particularly, in our case we have shallow indentation in the surface. A 532 nm laser incident beam with spot size <1 µm is precisely subjected to the indents. A low laser intensity was chosen to avoid laser damage to the sample. Confocal Raman spectroscopy enables a direct measurement of the local distribution of mechanical stress and strain in samples. Any extra phases can be detected due to its high sensitivity to modifications in the equilibrium distance between atoms.

#### TEM & FIB imaging

In order to observe the deformation process beneath the indenter tip, as well as the shear plasticity, a bright field cross sectional transmission electron microscopy (XTEM) images are carried out on selected indents using FEI Tecnai TF-20 FEG/TEM operated at 200 kV in bright-field (BF). The TEM-ready sample was prepared using the *in-situ* FIB lift out technique on a FEI Dual Beam FIB/SEM. The sample was capped with sputtered C and e-Pt/I-Pt prior to milling to protect the surface of the area of interest.

## References

[1] Cho S, Kang IM, Kim KR, Park BG, Harris JS (2013). Silicon-compatible high-hole-mobility transistor with an undoped germanium channel for low-power application. Appl. Phys. Lett..

[2] Dushaq G, Nayfeh A, Rasras M (2017). Metal-germanium-metal photodetector grown on silicon using low temperature RF-PECVD. Optics Express.

[3] Nayfeh A, Chui CO, Yonehara T, Saraswat KC (2005). Fabrication of high-quality p-MOSFET in Ge grown heteroepitaxially on Si. IEEE Electron Device Letters.

[4] Dushaq G, Rasras M, Nayfeh A (2017). Germanium MOS capacitors grown on Silicon using low temperature RF-PECVD. Journal of Physics D: Applied Physics.

[5] Khandelwal S, Duarte JP, Chauhan YS, Hu C (2014). Modeling 20-nm Germanium FinFET with the Industry Standard FinFET Model. IEEE Electron Device Letters.

[6] Pillarisetty R (2011). Academic and industry research progress in germanium nanodevices. Nature.

[7] Kolahdouza M, Farniya A, Benedetto LD, Radamson HH (2010). Improvement of infrared detection using Ge quantum dots multilayer structure. Appl. Phys. Lett..

[8] Soref, R. Mid-infrared photonics in silicon and germanium. *Nature Photonics* **4** (2010).

[9] Rössner B, Chrastina D, Isella G, von Känel H (2004). Scattering mechanisms in high-mobility strained Ge channels. Appl. Phys. Lett..

[10] Leea ML, Fitzgerald EA (2005). Strained Si, SiGe, and Ge channels for high-mobility metal-oxide-semiconductor field-effect transistors. Journal of Applied Physics.

[11] Liu J, Sun X, Camacho-Aguilera R, Kimerling LC, Michel J (2010). Ge-on-Si laser operating at room temperature. Optics Letter.

[12] Liu J, Kimerling LC, Michel J (2012). Monolithic Ge-on-Si lasers for large-scale electronic–photonic integration. Semicond. Sci. Technol..

[13] Oliver WC, Pharr GM (1992). An Improved technique for determining hardness and elastic modulus using load and displacement sensing indentation experiments. Journal of Material Research.

[14] Han SM, Saha R, Nix WD (2006). Determining hardness of thin film in elastically mismatched film-on-substrate systems using nanoindentation. Acta Materialia.

[15] Dietiker M, Nyilas RD, Solenthaler C, Spolenak R (2008). Nanoindentation of single-crystalline gold thin films: Correlating hardness and the onset of plasticity. Acta Materialia.

[16] Alkhatib A, Nayfeh A (2013). A Complete Physical Germanium-on-Silicon Quantum Dot Self-Assembly Process. Scientific Reports.

[17] Taylor C (2008). Direct self-assembly of quantum structures by nanomechanical stamping using probe tips. Nanotechnology.

[18] Kitayama D, Yoichi T, Suda Y (2006). Artificially positioned multiply stacked Ge dot array. Thin Solid Films.

[19] Yan J (2006). Load effects on the phase transformation of single-crystal silicon during nanoindentation tests. Materials Science and Engineering A.

[20] Jang J, Lance MJ, Wen S, Tsui TY, Pharr GM (2005). Indentation-induced phase transformations in silicon: influences of load, rate and indenter angle on the transformation behavior. Acta Materialia.

[21] Bradby JE, Williams JS, Wong-Leung J (2002). Nanoindentation-induced deformation of Ge. Appl. Phys. Lett..

[22] Deshmukh S (2014). Phase transformation pathways in amorphous germanium under indentation pressure. Journal of Applied Physics.

[23] Williams JS (2013). Hexagonal germanium formed via a pressure-induced phase transformation of amorphous germanium under controlled nanoindentation. Phys. Status Solidi RRL.

[24] Oliver DJ, Bradby JE, Williams JS, Swain MV, Munroe P (2009). Rate-dependent phase transformations in nanoindented germanium. Journal of Applied Physics.

[25] Olivera DJ, Bradby JE, Williams JS (2007). Giant pop-ins and amorphization in germanium during indentation. Journal of Applied Physics.

[26] Jang J, Lance MJ, Wen S, Pharr GM (2005). Evidence for nanoindentation-induced phase transformations in germanium. Appl. Phys. Lett..

[27] Lai, M., Zhang, X. & Fang, F. Nanoindentation-induced phase transformation and structural deformation of monocrystalline germanium: a molecular dynamics simulation investigation. *Nanoscale Res Lett*. **8**(1), 353, 201 (2013). 10.1186/1556-276X-8-353 PMC3765234 23947487

[28] Lloyd SJ, Molina-Aldareguia JM, Clegg WJ (2001). Deformation under nanoindents in Si. Ge and GaAs examined using transmission electron microscopy. J. Mater. Res..

[29] Nelmes RJ, McMahon MI, Wright NG, Allan DR, Loveday JS (1993). Stability and crystal structure of BCS germanium. Physical Review B.

[30] Huston LQ, Kiran MSRN, Smillie LAJ, Williams S, Bradby JE (2017). Cold nanoindentation of germanium. Appl. Phys. Lett..

[31] Domnich V, Gogotsi Y, Dub SN (2000). Effect of phase transformations on the shape of the unloading curve in the nanoindentation of silicon. Appl. Phys. Lett..

[32] Cheong WCD, Zhang LC (2000). Molecular dynamics simulation of phase transformations in silicon monocrystals due to nano indentation. Nanotechnology.

[33] Sophia, P. J. *et al*. Nanoindentation Studies of Metal Organic Vapor Phase Epitaxy Grown Ge/Si Heterostructures. *Energy and Environment Focus* **2** (4), 1, 85–89 (2013)

[34] Oliver DJ, Bradby JE, Williams JS, Swain MV, Munroe P (2008). Thickness-dependent phase transformation in nanoindented germanium thin films. Nanotechnology. Nanotechnology.

[35] Manika I, Maniks J (2008). Effect of substrate hardness and film structure on indentation depth criteria for film hardness testing. J. Phys. D: Appl. Phys..

[36] Menčik J, Munz D, Quandt E, Weppelmann ER (1997). Determination of elastic modulus of thin layers using nanoindentation. J. Mater. Res..

[37] Tunvisut K, O’Dowd NP, Busso EP (2001). Use of scaling functions to determine mechanical properties of thin coatings from microindentation tests. Int. J. Solids Struct..

[38] Gamonpilas C, Busso EP (2004). On the effect of substrate properties on the indentation behavior of coated systems. Materials Science and Engineering A.

[39] Dushaq G, Rasras M, Nayfeh A (2017). Low temperature deposition of germanium on silicon using radio frequeny chemical vapor deposition. Thin Solid Film.

[40] Fischer-Cripps, A. C. *Nanoindentation*, 3th edition, (Springer-Verlag, New York, 0941–5122, 2004).

[41] Minomura S, Drickamer HG (1962). Pressure induced phase transitions in silicon, germanium and some III–V compounds. J. Phys. Chem. Solids.

[42] Jamieson JC (1963). Crystal Structures at High Pressures of Metallic Modifications of Silicon and Germanium. Science.

[43] Johnson BC (2013). Evidence for the R8 Phase of Germanium. PRL.

[44] Kosai K, Huang H, Yan J (2017). Comparative study of phase transformation in single-crystal germanium during single and cyclic Nano-indentation. Crystals.

[45] Brazhkin VV, Lyapin AG, Popova SV, Voloshin RN (1992). Solid-phase disordering of bulk Ge and Si samples under pressure. JETP Lett..

[46] Dushaq G, Rasras M, Nayfeh A (2017). Hydrogen-Induced Crystallization of Germanium Films at Low Temperature Using an RF-PECVD Reactor. ECS Trans..

[47] Jian, S. R. Mechanical Deformation Induced in Si and GaN Under Berkovich Nanoindentation. Nano scale research letter 3, 6–13, and (2008).

[48] Lin YH, Chen TC, Yang PF, Jian SR, Lai YS (2007). Atomic-level simulations of nanoindentation-induced phase transformation in mono-crystalline silicon. Appl. Surf. Sci..

[49] Hay J, Agee P, Herbert E (2010). Continuous Stiffness measurements during instrumented indentation testing. Experimental Techniques.

[50] Gao FM (2003). Hardness of Covalent Crystals. Phys. Rev. Lett..

[51] Oliver DJ (2009). Nanoindentation of ion-implanted crystalline germanium. Physical Review B.

[52] Gadelraba, K. R., Chiesa, M. & Bonilla, F. A implication of the idea of effective tip shape on nanoindentation unloading curves: AFM measurements and FE simulation. *Journal of Materials Research* **27**(1) (2012).

[53] Pharr G, Bolshakov A (2002). Understanding nanoindentation unloading curves. J. Mater. Res..

[54] Chudoba T, Jennett N (2008). Higher accuracy analysis of instrumented indentation data obtained with pointed indenters. J. Phys. D Appl. Phys..

[55] Bolshakov, A., Oliver, W. C. & Pharr, G. M. *An explanation for the shape of nanoindentation unloading curves based on finite element simulation, in Thin Films: Stresses and Mechanical Properties*. p. 675 (Mater. Res. Soc. Symp. Proc. 356, Pittsburgh, PA, 1995).

[56] Dushaq, G., Nayfeh, A. & Rasras, M. Passivation of Ge/high-κ interface using RF Plasma nitridationSemicond. *Sci. Technol*. **33**, 015003, 9pp (2018).

